# The emergence of insecticide resistance in central Mozambique and potential threat to the successful indoor residual spraying malaria control programme

**DOI:** 10.1186/1475-2875-10-110

**Published:** 2011-05-02

**Authors:** Ana P Abilio, Immo Kleinschmidt, Andrea M Rehman, Nelson Cuamba, Varsha Ramdeen, David S Mthembu, Sarel Coetzer, Rajendra Maharaj, Craig S Wilding, Andrew Steven, Marlize Coleman, Janet Hemingway, Michael Coleman

**Affiliations:** 1 National Institute of Health, Av. Eduardo Mondlane/Salvador Allende, P.O. Box 264, Maputo, Mozambique; 2 London School of Hygiene & Tropical Medicine, Keppel Street, London WC1E 7HT, UK; 3 Malaria Research Programme, MRC, 491 Ridge Road, Overport, Durban, RSA; 4 Liverpool School of Tropical Medicine, Pembroke Place, Liverpool. L3 5QA, UK

## Abstract

**Background:**

Malaria vector control by indoor residual spraying was reinitiated in 2006 with DDT in Zambézia province, Mozambique. In 2007, these efforts were strengthened by the President's Malaria Initiative. This manuscript reports on the monitoring and evaluation of this programme as carried out by the Malaria Decision Support Project.

**Methods:**

Mosquitoes were captured daily through a series of 114 window exit traps located at 19 sentinel sites, identified to species and analysed for sporozoites. *Anopheles *mosquitoes were collected resting indoors and tested for insecticide resistance following the standard WHO protocol. Annual cross sectional household parasite surveys were carried out to monitor the impact of the control programme on prevalence of *Plasmodium falciparum *in children aged 1 to 15 years.

**Results:**

A total of 3,769 and 2,853 *Anopheles gambiae s.l*. and *Anopheles funestus*, respectively, were captured from window exit traps throughout the period. In 2010 resistance to the pyrethroids lambda-cyhalothrin and permethrin and the carbamate, bendiocarb was detected in *An. funestus*. In 2006, the sporozoite rate in *An. gambiae s.s*. was 4% and this reduced to 1% over 4 rounds of spraying. The sporozoite rate for *An. funestus *was also reduced from 2% to 0 by 2008. Of the 437 *Anopheles arabiensis *identified, none were infectious. Overall prevalence of *P. falciparum *in the sentinel sites fell from 60% to 32% between October 2006 and October 2008.

**Conclusion:**

Both *An. gambiae s.s*. and *An. funestus *were controlled effectively with the DDT-based IRS programme in Zambézia, reducing disease transmission and burden. However, the discovery of pyrethroid resistance in the province and Mozambique's policy change away from DDT to pyrethroids for IRS threatens the gains made here.

## Background

The Roll Back Malaria, United Nations Millennium Development Goals and World Health Assembly universal access and coverage targets for malaria prevention and treatment have been established to reduce disease transmission. To meet these targets malaria control interventions are now being scaled up [1,2].

To successfully control malaria, programmes must use current tools efficiently. In moderate to high transmission areas this requires the combination of effective vector interventions, either indoor residual spraying of insecticides (IRS) and/or insecticide-treated nets (ITNs), with effective drug treatment. To achieve this, continuous surveillance, monitoring and evaluation (M & E) need to be integrated into the malaria control programme. There are very few examples of good malaria M & E, which include parasitological, disease transmission and entomological data assessment. These include Bioko island [3,4], the cross border Lubombo Spatial Development Initiative (LSDI) [5], Eritrea [6] and Zambia [7].

In Mozambique, the National Malaria Control Programme (NMCP) used DDT for IRS before a change in policy in 1993, when the pyrethroid lambda-cyhalothrin was introduced in the southern part of the country. With the discovery of *Anopheles funestus *resistant to pyrethroids in South Africa [8], the LSDI implemented an insecticide resistance monitoring programme in southern Mozambique that showed in 1999 that both *An. funestus *and *Anopheles arabiensis *were resistant to pyrethroids [9,10] due to elevated p450s [11]. This resulted in an informed insecticide policy change to the carbamate bendiocarb for IRS [5]. Bendiocarb was sprayed bi-annually in southern Mozambique until 2005, when, due to the high economic costs associated with this insecticide, an operational change was made back to DDT. No resistance has yet been detected to DDT in Mozambique.

In 2005 the U.S. Government initiated the President's Malaria Initiative (PMI) [12], which supported the rapid scale-up of malaria prevention and treatment in 15 countries in Africa, including Mozambique. In central Mozambique, an IRS programme was initiated in six districts in Zambézia province. Previously, the NMCP had carried out vector control with sporadic IRS and fogging between 1995 and 2003 in this area. In 2005, the NMCP resumed IRS in Zambézia in three districts, using DDT. Limited expansion of activities occurred in 2006 to cover 5 districts and this effort was strengthened in 2007 by PMI. The IRS was focused on densely populated areas using DDT or lambda-cyhalothrin, the latter being applied on structures not suitable for DDT (i.e. finished or painted walls). In 2009, pyrethroids were the sole class of insecticides purchased for IRS, although all remaining stocks of DDT were sprayed during that year [13].

Zambézia province was used to pilot and refine a new Malaria Decision Support System (MDSS) [14], which was implemented in 19 sentinel sites in Zambézia province in 2006 and served to evaluate and monitor the interventions described above. The objective of this work was to determine whether the system, which was continuously improved during the course of the study, could be deployed in a resource poor setting, with low level infrastructure and limited human capacity. The utility to control programme managers of an integrated surveillance system providing real time monitoring through a number of performance and impact indicators, was clearly demonstrated.

## Methods

### Study site and insecticide usage

**Figure 1:**
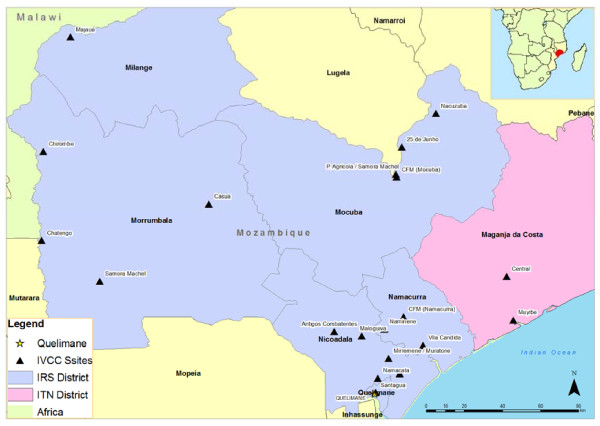
**Map showing the location of sentinel sites in Zambézia province, Mozambique**.

In 2006, 19 sentinel sites were established in six districts in Zambézia province, Mozambique. IRS with DDT (Hindustan Insecticide limited, New Delhi, India) was carried out in Oct/Nov 2006 and subsequently each year in all districts except for Maganja da Costa, where LLINs (both Olyset, Sumitomo Chemical and Permanent, Vestergaard Frandsen) were distributed from 2009 (Figure 1). Entomological surveillance and cross-sectional malaria indicator surveys were carried out at all 19 sites.

### Insecticide resistance testing

Indoor resting blood fed adult female *Anopheles gambiae s.l *and *An. funestus *for insecticide resistance bioassays were collected between hours 06.00-10.00 in houses using an aspirator during the period Oct 2006 to May 2010. Until 2009, wild caught mosquitoes were used for insecticide susceptibility assays following the WHO insecticide susceptibility test protocol (WHO 1998). Assays carried out on wild-caught mosquitoes did not allow for standardisation in age, physiological state or pre-exposure to insecticides. After the establishment of an insectary in 2009, wild-caught female mosquitoes were transported to the laboratory, kept in individual oviposition tubes with access to 10% sucrose and allowed to lay eggs. Each family was reared separately through to 1-3 day old F1 adults at 26°C +/- 2°C and 70-80% relative humidity allowing standardisation of age physiological state and testing conditions. Families were mixed prior to testing to avoid bias from isofemale lines where offspring may all be genetically similar. Between five and 25 adult mosquitoes were exposed to insecticide treated or control papers, impregnated with the carrier oil alone, for 1 hour and then transferred to holding tubes with access to 10% sugar solution for 24 hours before the percentage mortality was determined. The insecticides tested were, deltamethrin (0.05%), lambda-cyhalothrin (0.05%), bendiocarb (0.01%), and DDT (4%). All papers were supplied by WHO.

### Species abundance and sporozoite rates

Prior to the initiation of IRS in 2006 a window exit trap was installed in six houses at each sentinel site with the home-owner's permission. Home-owners were trained to empty the traps daily into pre-labelled specimen jars containing isopropanol and complete checklists indicating the nights for which the traps were checked.

### Species identification

Wild-caught females were morphologically identified as belonging to the *An. gambiae s.l*. complex or *An. funestus s.l*. group [15,16]. Species identification within each complex was carried out by DNA-polymerase chain reaction on a sample of specimens [17,18].

### Sporozoite identification

DNA was extracted from the head and thorax of a subsample of mosquitoes using the Livak method [19] and infection with *Plasmodium *species was determined using a multiplex real time PCR [20].

### Household surveys

Cross sectional household surveys based on the malaria indicator survey of the Roll Back Malaria Monitoring and Evaluation Reference Group [21] were carried out at each sentinel site on a random sample of 140 children between 1 and 15 years of age. Households were selected from strata formed by dividing sentinel sites into quadrants from which participants were selected, to ensure the greatest geographical spread within the site. Diagnostic tests with ICT™ malaria rapid tests (ICT, Global Diagnostics, Cape Town, South Africa.) were used to assess the *Plasmodium falciparum *infection status of each of the sampled children. Participants who tested positive were offered treatment with Coartem^® ^(Novartis) (artemether and lumefantrine) according to Mozambique's national malaria treatment guidelines.

Household surveys were carried out prior to the initiation of IRS in October 2006 and subsequently in the same month in 2007 and 2008. The sentinel site-specific sample size was calculated to provide evidence at the 5% significance level of an absolute reduction in *P. falciparum *prevalence of 20% following the intervention. Prevalence and 95% confidence intervals (CI) for each sentinel site were estimated taking account of clustering by sentinel site using the statistical software package STATA (StataCorp LP. Stata Statistical Software: Release 10. College Station, TX, USA.).

Sentinel sites were considered the primary sampling unit. Logistic regression, allowing for complex survey designs, was performed to estimate the mean effect of the intervention on prevalence compared to baseline prevalence of infection in different years.

### Ethics

Ethics was approved for this work by the Ministry of Health, Mozambique Reg: 3622/IMS-2/DNS/06.

## Results

### Vector abundance and transmission

**Figure 2:**
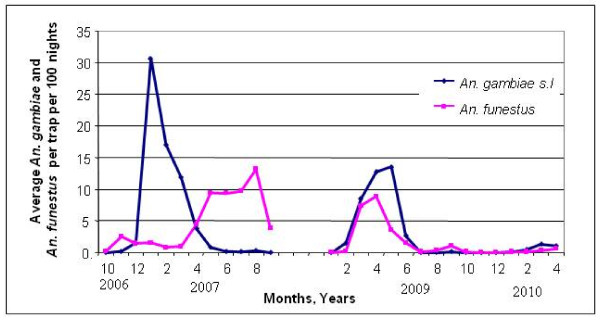
***An. gambiae s.l*. and *An. funestus *species abundance from all 19 sentinel sites combined**. Collections did not occur 09 2007 to 01 2009 due to logistical problems.

Over the 44 month study period, a total of 6,622 anophelines were collected from 114 window exit traps that operated continuously. Three thousand seven hundred and sixty nine were morphologically identified as *An. gambiae s.l*. and 2,853 as *An. funestus*. Of these, 905 *An. gambiae s.l*. and 946 *An. funestus *were further identified to species level and tested for sporozoites (Table 1). *Anopheles gambiae *and *An. arabiensis *were the only two members of the *An. gambiae *complex and *An. funestus s.s *the only member of the *An. funestus *group to be identified. *Anopheles gambiae s.s*. was the predominant species in all spray round periods from the window exit trap collections (Figure 2, Table 1).

**Table 1:** Abundance, sporozoite rate and relative transmission index of specimens collected over a two years period, at all sentinel sites in Zambézia province, 2006-2010.

	Period 1	Period 2
		
	2006 to 2007	2009 to 2010
*An. gambiae s.l*		
No Caught	2304	1465
No analyzed for species identification	456	449
Proportion *An. gambiae s.s *(%)	91	63
Proportion *An. arabiensis *(%)	5	22
		
*An. Arabiensis*		
No estimated	115	322
No per trap per 100 nights	0.058	0.2
Sporozoite rate (%)	0 (n = 24)	0 (n = 98)
Transmission index	NA	NA
Transmission index relative to baseline	1	NA
		
*An. gambiae s.s*		
No estimated	2097	923
No per trap per 100 nights	0.997	0.59
Sporozoite rate (%)	4.096 (n = 415)	1.053 (n = 285)
Transmission index	0.0486	0.0062
Transmission index relative to baseline	1	0.13
		
*An. funestus s.s*		
No Caught	1997	856
No per trap per 100 nights	1.59	0.19
Sporozoite rate (%)	2.272 (n = 660)	0 (n = 272)
Transmission index	0.036	NA
Transmission index relative to baseline	1	NA

NA = Not Applicable

Between 2006 and 2010 there was a steady decline in the abundance of *An. gambiae s.l*. and *An. funestus *with the estimated number of *An. gambiae *per window trap per 100 nights falling from 10 to 0.59 and *An. funestus *declining from 1.59 per window trap per 100 nights to 0.2. A spike in *An. funestus *abundance (May to Oct 2007) occurred only in Maganja da Costa where control operations were minimal in 2007. Prior to the LLIN distribution in Maganja da Costa in 2008 the abundance of *An. gambiae s.l *and *An. funestus *was 52.2 and 88.6 per window trap per 100 nights respectively. This number was reduced dramatically to 2.7 in 2009 and 1.6 in 2010 for *An. gambiae s.l*. and to zero for *An. funestus *for both 2009 and 2010 (data not tabulated). The proportion of *An. arabiensis *increased from 5% to 15% of the total *An. gambiae s.l*. complex caught.

In the period 2006 to 2007, combined sporozoite rates across all IRS districts were 4.1% and 2.3% for *An. gambiae s.s *and *An. funestus *respectively. After four rounds of IRS this was reduced to 1% for *An. gambiae s.s *and to zero for *An. funestus *in the 2009 to 2010 period (Table 1). No infective *An. arabiensis *were identified pre- or post-intervention.

The relative transmission index, defined as the ratio of infective anopheline abundance, relative to the baseline period of 2006/7 was 0.13 for *An. gambiae s.s*. for the follow-up period 2009/10. After three spray rounds no infected *An. funestus s.s *were caught, so no transmission index could be calculated.

### Insecticide resistance

A total of 3,664 *Anopheles *were tested for insecticide resistance between April 2006 and April 2010, of which 1,471 were wild-caught adult mosquitoes prior to the establishment of an insectary, and 1,786 were F1 generation mosquitoes from 105 families after an insectary was established. After the implementation of IRS in Zambézia province, the number of mosquitoes collected in the field was significantly reduced making collections difficult for resistance testing and resulting in only 13 of the 19 sites being tested (Table 2).

**Table 2:** WHO susceptibility test result on wild-caught adult female mosquitoes from 2006 to 2008 and one-three day old female F1 generation from 2010, for *Anopheles funestus *and *Anopheles gambiae s.l *from different localities of Zambézia province Mozambique.

	2006 to 2008									
	
Locality	Bendiocarb (0.01%)		DDT (4%)		Deltamethrin (0.05%)		Lamba cyhalothrin (0.05%)		Permethrin (0.75%)	
	% M	n	% M	n	% M	n	%M	n	%M	n
*An. funestus *										
Quelimane					100	28	100	50		
A. combatentes			100	25	100	25				
Namacurra			100	20						
Maganja da Costa	100	22	100	60	100	85	100	74		
Muibi	100	28	100	45	100	5	100	11		
CFM-Mocuba			100	59	100	78				
Posto Agricola			100	209	100	121				
25 de Junho			100	79	100	31				
Mugeba										
Majaua										
										
*An. gambiae s.l*										
Quelimane	100	91			100	15	100	130	100	4
Nicoadala	100	60					100	56		
Central			100	30	100	30				
Muibi										
Nacuzuba										

	2010									
	
*An. funestus*										
Mugeba	93.5	229	100	224			76.2	234	99.4	197
Majaua	84.5	207	100	193			82.9	159	100	193
										
*An. gambiae s.l*.										
Muibi			100	29			100	14		
Nacuzuba			100	44			100	43	100	20

No resistance to any of the insecticides tested was detected in the province prior to 2010. In 2010, resistance to the carbamate, bendiocarb and the pyrethroid, lambda-cyhalothrin was detected in F1 generation *An. funestus *from Majaua (84.5% and 82.9% mortality respectively) and Mugeba (93.5% and 76.2% mortality respectively). A low level of survival to the pyrethroid permethrin was also detected in *An. funestus *from Majaua (99.4% mortality) which requires further investigation (Table 2). No resistance was detected in *An. gambiae*. All species remained fully susceptible to DDT.

### Malaria prevalence

**Figure 3:**
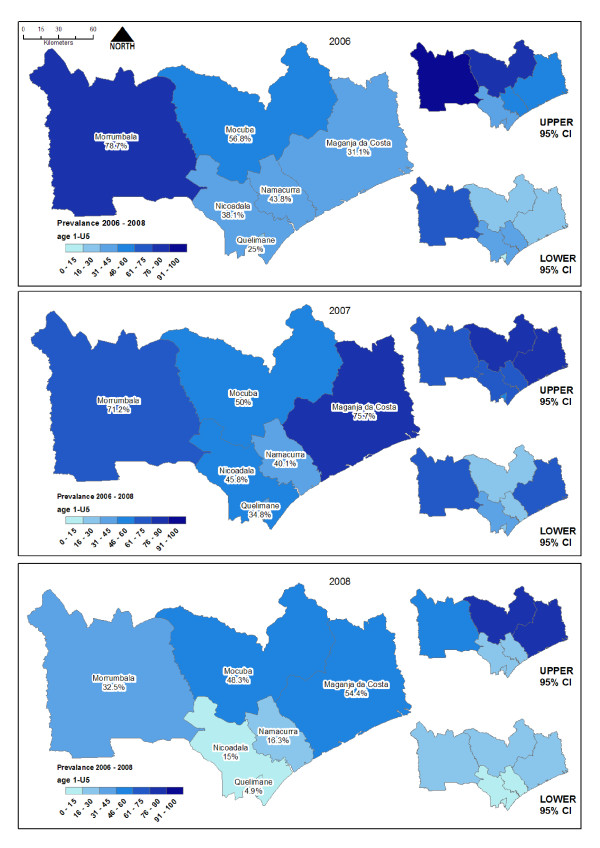
**Temporal change in prevalence of *P. falciparum *in children 1 to 15 years of age between 2006 and 2009**.

A total of 4,864 children between one and 15 years of age were surveyed in 2006 for *P. falciparum *infection. Follow-up surveys were carried out in 2007 and 2008 involving 5,314 and 5,258 children respectively from the same sentinel sites. The refusal rate was not recorded but was negligible. Combined prevalence of infection with *P. falciparum *across all 19 sites surveyed in October 2006 was 52.3% (CI = 40.62-63.6), ranging from 24.8% (CI = 15.32-37.46) in Quelimane, the urban provincial capital, to 77.2% (CI = 50.7-91.7) in Morrumbala, the most rural area in this study. In 2007 overall prevalence for the same 19 sites showed no significant difference at 60.4% (CI = 50.54-69.56), however, for Maganja da Costa there was evidence of an increase in prevalence from 39% (CI = 22.7-58.2) to 76.1% (CI = 61.8-86.3, P < 0.05). In 2008 there was strong evidence of a reduction in combined prevalence for the region to 32% (CI = 22.51-42.03) (Figure 3, Additional file 1), with the most significant reductions occurring in the more urban sites located in Nicoadala and Quelimane.

IRS coverage reached less than 50% of houses in the six districts targeted for spraying in 2007 due to limited resources. From 2008 the IRS programme was strengthened by PMI and IRS reached >80% coverage of the 502,000 houses in the six target districts, comprising approximately 2 million persons.

## Discussion

The success of the IRS-based LSDI programme in southern Mozambique [3] and the increase in malaria cases in northern Mozambique prompted the MOH and the NMCP to re-initiate a comprehensive vector control programme in Zambézia province. The MDSS project was embedded within the national programme, and demonstrated the feasibility and potential benefit of evaluating vector control interventions even in very resource poor settings. Intervention coverage and its impact on malaria vectors and on community prevalence of malarial infection were monitored as indicators of progress.

*Anopheles gambiae s.s *and *An. funestus *were the dominant malaria vector species in Zambézia province, where *An. gambiae s.s*. was predominant. This confirms results from previous studies of *Anopheles *distribution in northern Mozambique [22]. Although *An. arabiensis *was detected in this area, it was in low abundance with no sporozoite positive specimens found, indicating low transmission potential.

Mosquito species abundance changes naturally during annual cycles, depending on climatic variables [15,23]. Abundance of both *An. gambiae s.s *and *An. funestus *displayed seasonal variations, but were present throughout the year. *An. gambiae *peaked in January to May, at the height of the rainy season, whilst *An. funestus *was highest in April to August in 2007, towards the end of the rainy season, thus prolonging the malaria transmission season. However, in 2009, *An. funestus *peaked at the same time as *An. gambiae*. With this potential pattern of vector abundance the vector control interventions need to remain effective for a duration of eight months each year. This requires either an insecticide with a long residual half-life, such as DDT, or spraying multiple rounds each year.

After three rounds of IRS with DDT a dramatic impact on vector populations was observed. This was similar to that reported for other IRS programmes, including the LSDI programme in southern Mozambique [5] and Bioko, Equatorial Guinea [3], where vector densities declined when effective insecticides were applied. In Zambézia, abundance of the vectors fell significantly from 1 to 0.6 and 1.6 to 0.2 per exit trap per 100 nights for *An. gambiae *and *An. funestus *respectively. This corresponded with a reduction in the sporozoite rates from 4% to 1% in *An. gambiae *and 2% to 0% in *An funestus*. While remaining low, numbers of *An. arabiensis *collected after the interventions doubled, but as none of the samples tested positive for sporozoites, this species appears to have little or no involvement in malaria transmission. However, since *An. arabiensis *is known to be an effective malaria vector in other parts of Mozambique [5,24,25] and elsewhere, it is essential that this species continues to be monitored.

The vector control intervention has impacted on disease in the human population, as measured through prevalence surveys over three spray seasons. In the first year, IRS was carried out by the NMCP in Oct/Nov with limited resources. Only 50% of targeted houses were sprayed in each district. There was no measurable impact on disease prevalence in IRS sites after this intervention. In 2007 PMI support for the NMCP in this area was initiated, and IRS coverage increased to >80%, the rate recommend for effective IRS by WHO [26]. After this intervention there was a significant reduction in *P. falciparum *prevalence in children aged between one and 15 years in all IRS districts except Mocuba, where IRS had very little impact.

LLINs provide personnel protection, and with high coverage also offer community protection [27,28]. Data from Maganja da Costa (Additional file 1) show that a substantial decrease in parasite prevalence in the human population was associated with LLIN distribution in 2008. In this area, the abundance of *An. gambiae *and *An. funestus *declined from 52 and 88 per window trap per 100 nights to just 1.6 and 0 respectively. In 2006-2007, the rainfall was exceptionally high in this district leading to higher than usual rice cultivation in 2007. This may have resulted in increased vector abundance in the sentinel sites and increased transmission, which is reflected in the significant increase in prevalence in the 2007 survey. Since 2007, there has been limited rainfall, resulting in no rice cultivation. Both changes in rainfall pattern [29,30] and alteration in agricultural practises [31] will impact on the vector population, in this case to the advantage of malaria control. These severe climatic changes in Maganja da Costa make it difficult to assess the true impact of the LLIN distribution in this area over such a short time. Although not as severe, a climatic change was documented throughout the study area, and needs to be considered when interpreting the impact of interventions.

Prior to the detection of pyrethroid resistance in *An. funestus *in 2010 in sentinel sites in Mocuba, no insecticide resistance had been detected in the malaria vectors in this region to any insecticide. A low frequency of resistance may have been masked, as WHO discriminating doses are set at double the insecticide dose that gives 100% mortality of the least susceptible anopheline mosquitoes, making them good indicators of resistance only when resistance levels rise significantly in the mosquito population. Prior to 2010, and the establishment of an insectary in Zambézia, all resistance assays were carried out on wild-caught mosquitoes. This potentially affected resistance testing, as mosquitoes were not standardised for age, physiological state or pre exposure to insecticides. No DDT resistance was detected in the province. The detection of both bendiocarb and pyrethroid resistance at the same time, an unusual pattern of resistance, but one found in 2000 in southern Mozambique, thought initially to be due to cross resistance of elevated monooxygenase [11] but later data showed both elevated monooxygenase and altered acetylcholineesterase, a major mechanism of carbamate resistance, in *An. funestus *populations [32] suggests that this resistance may have spread.

Monitoring insecticide resistance mechanisms is an integral component of resistance management, allowing for informed decisions on insecticide choice and resistance management [33], as a number of mechanisms may give rise to different cross resistance patterns [34,35]. Within the national programme over this time period it was not possible to monitor for resistance mechanisms, due to the lack of a cold chain to a suitable laboratory. New developments in molecular techniques should make this possible in the future [36,37]. However, as the resistance pattern observed in *An. funestus *here is very distinct, and similar to that previously detected in southern Mozambique [11,32,38], with resistance to both carbamates and pyrethroids segregating together it is likely that this resistance has not arisen *de novo *in central Mozambique. This has been shown to be the result of increased levels of monooxygenases and acetylcholinesterase in the same population [32,38].

Currently Mozambique national policy has been to use lambda-cyhalothrin instead of DDT for IRS. The former was used in the 2009 spray round even though pyrethroid resistance has now been detected in Zambézia and previously in southern Mozambique [32,38-40].

In 1996, South Africa altered its insecticide of choice from DDT to the pyrethroid deltamethrin. After pyrethroid resistance was selected the recorded malaria cases increased more than six fold from 1995 to 1999. During this period there was also an increase in malaria drug resistance [41], but entomological surveys showed that *An. funestus*, which had previously been eliminated by DDT, had re-established in South Africa [8] from southern Mozambique due to the protection against IRS acquired through pyrethroid resistance. With the re-introduction of DDT [42] combined with the introduction of an effective drug [41] malaria control was once again successful in South Africa.

The recently detected low level pyrethroid resistance in *An. funestus *in Zambézia province reported here for the first time, and in neighbouring Likoma Island, Malawi, that is close to Zambezia [43], is likely to become operationally significant and should be closely monitored, as already there are anecdotal reports of *An. funestus *resting in pyrethroid sprayed houses and an increase in malaria cases in Zambézia province.

## Conclusion

The IRS-based vector control programme has had a successful impact on the malaria burden in Zambézia province. The effectiveness of IRS vector control with pyrethroids is at risk from resistance to this class of insecticide, which has already been detected in multiple locations in Mozambique. It is therefore important that a high level of vigilance is maintained through continued high quality entomological and epidemiological surveillance in this region so that corrective policy changes can be implemented before insecticide resistance leads to a public health emergency. This study demonstrates that the Malaria Decision Support System [44] provides an appropriate integrated platform for conducting such surveillance.

## Competing interests

The authors declare that they have no competing interests.

## Authors' contributions

All authors have read and approved the final manuscript. APA-carried out the entomological investigations and analysis and drafted the manuscript, IK and AR carried out all epidemiological analysis, helped interpret the results and contributed to writing the manuscript, NC-supported entomological work in country, VR-carried out diagnostic tests on mosquitoes from window exit traps, DM-supported field work, SC-carried out all GIS, RM-assisted in design of programme, CW and AS established insecticide resistance in the field, MC-carried out conceptual design and development of MDSS, JH-input into the writing of the manuscript, MC-was responsible for the overall project.

## Supplementary Material

Additional file 1 **Prevalence of infection**. Prevalence of infection with *P. falciparum *in children 1 to <15 years of age, by districts, observed during household surveys in 2006, 2007 and 2008 in Zambézia province, Mozambique. Click here for file
