# Supplementary material for: The emergence of insecticide resistance in central Mozambique and potential threat to the successful indoor residual spraying malaria control programme
Source: Malar J. 2011 May 2;10:110. doi: 10.1186/1475-2875-10-110 (PMC3096596; doi:10.1186/1475-2875-10-110)
Supplement: Additional file 1 — Prevalence of infection. Prevalence of infection with P. falciparum in children 1 to <15 years of age, by districts, observed during household surveys in 2006, 2007 and 2008 in Zambézia province, Mozambique. [file 1475-2875-10-110-S1.DOCX]

Additional file: Prevalence of infection with *P. falciparum* in children 1 to <15 years of age, by districts, observed during household surveys in 2006, 2007 and 2008 in Zambézia province, Mozambique.

|  |  | October 2006 | |  | October 2007 | |  |  | October 2008 | |  |
| --- | --- | --- | --- | --- | --- | --- | --- | --- | --- | --- | --- |
|  |  | Prevalence of infection % (n) | 95% Confidence interval |  | Prevalence of infection % (n) | 95% Confidence interval |  |  | Prevalence of infection % (n) | 95% Confidence interval |  |
|  | District |  |  |  |  |  |  |  |  |  |  |
|  |  |  |  |  |  |  | *P* |  |  |  | *P* |
|  | Maganja da Costa | 39 (159) | [22.7-58.2] |  | 76.1 (289) | [61.8-86.3] | <0.001 |  | 52.5 (280) | [21.4-81.8] | 0.037* |
|  | Mocuba | 49.5 (551) | [22.3-77.1] |  | 55.5 (569) | [31.0-77.7] | 0.558 |  | 47.1 (558) | [22.6-73.1] | 0.407 |
|  | Morrumbala | 77.2 (565) | [50.7-91.7] |  | 76.3 (557) | [72.3-79.9] | 0.944 |  | 35.9 (529) | [22.3-52.3] | <0.001 |
|  | Namacurra | 45.1(388) | [33.4-57.4] |  | 47.1 (420) | [26.8-68.5] | 0.836 |  | 22 (419) | [13.0-34.6] | 0.003* |
|  | Nicoadala | 45.3 (656) | [39.3-51.3] |  | 58.2 (677) | [44.0-71.2] | 0.205 |  | 19.2 (708) | [14.3-25.3] | <0.001 |
|  | Quelimane | 24.8 (113) | [15.3-37.4] |  | 36.6 (145) | [25.7-48.9] | 0.132 |  | 8.9 (135) | [4.5-16.5 ] | <0.001 |
|  | All | 52.3 | [40.6-63.6] |  | 60.4 | [50.5-69.5] | 0.445 |  | 32 | [22.5-43.1] | 0.003* |
